# Editorial: Women in psychiatry 2024: Psychopharmacology

**DOI:** 10.3389/fpsyt.2025.1716714

**Published:** 2025-11-07

**Authors:** Rosana Camarini, Alfreda Stadlin, Reem K. Jan

**Affiliations:** 1 Department of Pharmacology, Institute of Biomedical Sciences, University of São Paulo, São Paulo, Brazil; 2 Hong Kong University of Science and Technology, Hong Kong, Hong Kong SAR, China; 3 College of Medicine, Mohammed Bin Rashid University of Medicine and Health Sciences, Dubai, United Arab Emirates

**Keywords:** editorial, psychopharmacology, mechanistic insights, innovation, equitable psychiatric care, real-world pharmacology, women in psychiatry

## Translating innovation into equitable psychiatric care

Women’s mental health remains an urgent frontier in psychiatry. Conditions such as depression, posttraumatic stress disorder, and metabolic complications of antipsychotic treatment disproportionately affect women, yet clinical research has often underrepresented them. The six contributions in this Research Topic, all led by women scientists, exemplify how rigorous research can advance both mechanistic understanding and practical solutions, while also drawing attention to equity, sex differences, and patient-centered care. These studies remind us that innovation in psychiatry is not only about developing new molecules, but about ensuring that therapeutic advances are relevant and safe for all.

## Mechanistic insights and novel targets

Psychopharmacology research continues to clarify pathways relevant to psychiatric illness. Marina Wolf’s pioneering work on glutamate-driven synaptic plasticity has been central to understanding addiction, particularly through her elucidation of α-amino-3-hydroxy-5-methyl-4-isoxazolepropionic acid (AMPA) receptor trafficking in the nucleus accumbens and its role in cue-induced drug seeking (1). Building on these insights, in a manuscript entitled “*AMPA/kainate receptor activation within the prelimbic cortex is necessary for incubated cocaine craving*”, Sánchez et al. investigated AMPA/kainate receptor activation in the prelimbic cortex during the incubation of cocaine craving. Their findings showed that AMPA/kainate receptor blockade prevented incubated craving during late withdrawal, but unexpectedly increased cue-induced seeking in females during early withdrawal. Although sex differences in the magnitude of craving were not detected, the work underscores the necessity of considering biological sex differences in addiction research.

In “*Phytochemicals in the treatment of patients with depression: a systemic review*”, Picheta et al. conducted a systematic review of phytotherapy for depression. Examining randomized controlled trials of *Crocus sativus*, *Hypericum perforatum*, *Lavandula angustifolia*, and *Curcuma longa*, they reported consistent antidepressant effects, partly mediated by anti-inflammatory and antioxidant pathways. With depression affecting women at nearly twice the rate of men (2), and often accompanied by comorbidities, such findings highlight the need for culturally adaptable and better-tolerated treatments that expand options beyond conventional pharmacotherapy.

## Real-world pharmacology

Even when pharmacological tools are well validated in clinical trials, their real-world use can differ significantly. Mora et al. in “*Lurasidone uses and dosages in Spain: RETROLUR, a real-world retrospective analysis using artificial intelligence*” analyzed electronic health records from Spanish hospitals in their study of lurasidone use (RETROLUR). Most patients initiating lurasidone were women with metabolic comorbidities and substantial prior antipsychotic exposure. Interestingly, most patients received relatively low doses compared to those tested in clinical trials, yet reported improvement in positive and negative symptoms, anxiety, and depression. These results highlight both the promise and limitations of lurasidone: favorable tolerability supports its real-world adoption, but low dosing may restrict its therapeutic potential.

The management of antipsychotic-induced weight gain (AIWG) is another pressing challenge. Fitzgerald et al. revisited the role of metformin, a World Health Organization essential medicine, in addressing this problem in the article “*Metformin in the management of antipsychotic-induced weight gain – why the ‘weight’?*”. Approaches such as antipsychotic dose reduction, discontinuation, or switching, yield only modest weight reductions, with uncertain clinical relevance (3, 4). Current guidelines typically recommend metformin only after lifestyle changes or switching fail. However, evidence suggests that postponing metformin initiation undermines its preventive potential, especially early in antipsychotic treatment. For women with severe mental illness who also face obesity—a dual stigma that compounds barriers to care—early metformin initiation represents a pragmatic, equitable, and low-cost intervention with potential to reduce long-term morbidity and mortality. This perspective calls for recalibrating guidelines to reflect patient trajectories more faithfully.

## Optimizing care through context and professional collaborations

The remaining two contributions underscore that effective treatments depend not only on the agent itself, but also on context, safety, and interdisciplinary collaboration. In “*Bringing MDMA-assisted therapy for PTSD to traditional healthcare systems: tending to set and setting*”, Perivoliotis et al. discuss the development of 3,4-methylenedioxymethamphetamine (MDMA)-assisted therapy for post-traumatic stress disorder (PTSD), highlight the importance of “set and setting”, i.e, the patient’s mental state and expectations, and the therapeutic environment. Even the most effective trauma-focused psychotherapies achieve remission in about half of patients. Thus, MDMA-assisted therapy may expand the therapeutic toolkit, but successful implementation requires clinician training, structured preparation, safe environments, and adaptation to healthcare system realities. For women, who experience higher PTSD rates and remain underrepresented in trials (5), such contextual safeguards are especially critical to ensure equitable access.

Finally, Wien et al. present a systematic review - “*Prevalence and solving strategies of drug-related problems in adult psychiatric inpatients - a systematic review*”- of drug-related problems (DRPs) in psychiatric inpatients, analyzing data from nearly 100,000 patients. Prescribing errors and drug–drug interactions emerged as the most frequent issues, with women particularly affected due to higher rates of polypharmacy and adverse events. The review underscores the pivotal role of clinical pharmacists integrated into multidisciplinary teams, not only in detecting DRPs but also in ensuring continuity of care through hospitalization and discharge. Specialized training, such as the Board-Certified Psychiatric Pharmacist credential, enhances their effectiveness, while digital tools like computerized order entry systems can complement (but not replace) individualized clinical expertise. Of particular importance is vigilance in vulnerable populations: for instance, pregnant women with hypertension and gestational diabetes frequently experience DRPs due to inadequate treatment effectiveness and higher rates of adverse effects (6).

Taken together, these six contributions underscore that progress in psychiatry requires advances across multiple dimensions: elucidating molecular mechanisms, rigorously evaluating both novel and traditional therapies, aligning guidelines with real-world practice, and embedding interventions within safe and supportive contexts. Importantly, each article also invites reflection on the role of gender in psychiatric care.
